# Promoter Proximal Pausing Limits Tumorous Growth Induced by the Yki Transcription Factor in *Drosophila*

**DOI:** 10.1534/genetics.120.303419

**Published:** 2020-07-30

**Authors:** Sanket Nagarkar, Ruchi Wasnik, Pravallika Govada, Stephen Cohen, L. S. Shashidhara

**Affiliations:** * Indian Institute of Science Education and Research (IISER), Pashan, Pune 411008; † Department of Cellular and Molecular Medicine, University of Copenhagen, 2200N, Denmark; ‡ Ashoka University, Sonepat, Haryana 131029, India

**Keywords:** tumorigenesis, *Drosophila*, Hippo pathway, promoter proximal pausing, transcription regulation in growth and cancer

## Abstract

Promoter proximal pausing (PPP) of RNA polymerase II has emerged as a crucial rate-limiting step in the regulation of gene expression. Regulation of PPP is brought about by complexes 7SK snRNP, P-TEFb (Cdk9/cycT), and the negative elongation factor (NELF), which are highly conserved from *Drosophila* to humans. Here, we show that RNAi-mediated depletion of *bin3* or *Hexim* of the 7SK snRNP complex or depletion of individual components of the NELF complex enhances Yki-driven growth, leading to neoplastic transformation of *Drosophila* wing imaginal discs. We also show that increased CDK9 expression cooperates with Yki in driving neoplastic growth. Interestingly, overexpression of CDK9 on its own or in the background of depletion of one of the components of 7SK snRNP or the NELF complex necessarily, and specifically, needed Yki overexpression to cause tumorous growth. Genome-wide gene expression analyses suggested that deregulation of protein homeostasis is associated with tumorous growth of wing imaginal discs. As both Fat/Hippo/Yki pathway and PPP are highly conserved, our observations may provide insights into mechanisms of oncogenic function of YAP—the ortholog of Yki in humans.

REGULATION of growth is arguably the most critical phenomenon that establishes size and shape of all tissues, organs, and overall body size in metazoan animals . It is also an important homeostatic process, failure of which is linked to diseases and disorders, particularly cancer in humans. Regulated growth is achieved by an intricate interplay between factors promoting growth (oncogenes) and those suppressing it (tumor suppressors).

Yorkie (Yki), the *Drosophila* ortholog of the Yes-Associated Protein 1 (YAP1), acts as a transcriptional cofactor that mediates the effects of the Hippo tumor suppressor pathway. The Hippo pathway is highly conserved from *Drosophila* to humans (Pan 2010). The Hippo (Hpo)/MST kinases and the Warts (Wts)/LATS kinases and their cofactors form kinase cassettes that directly phosphorylate Yki (YAP/TAZ) to regulate protein stability and activity (Zhao *et al.* 2011). Members of this pathway were initially found to limit tissue growth in *Drosophila* by limiting Yki activity (Huang *et al.* 2005; Dong *et al.* 2007). Consistent with this, YAP overexpression has been reported as a driver of tissue growth and cancer in a mouse model (Dong *et al.* 2007; Zanconato *et al.* 2015). In humans, the YAP1 locus was found to be amplified in different types of cancer (Overholtzer *et al.* 2006; Zender *et al.* 2006). These findings have sparked a great deal of interest in understanding of regulation of Yki/YAP function.

In *Drosophila*, Yki regulates expression of regulators of cell growth and survival such as *Diap1*, *dMyc*, *bantam*, etc. Targets of YAP in humans include the EGFR-ligand AREG as well as *CTGF*, *Cyr61* (Johnson and Halder 2014). While these target genes are necessary for growth induced by Yki/YAP activity, they are not sufficient to phenocopy effects of Yki/YAP. This indicates possibility of more regulators that are involved in Yki/YAP induced growth.

We have reported an *in vivo* screen in *Drosophila* (Groth *et al.* 2020), wherein we have identified a large number of genes, which, when depleted, enhanced growth induced by Yki and EGFR. More importantly, these genes function like classical tumor suppressors as, when downregulated in the background of overexpressed Yki or EGFR, we observed neoplastic growth. Among these, we identified a number of genes involved in the control of promoter proximal transcriptional pausing.

Promoter proximal pausing (PPP) of RNA polymerase (Pol) II has been identified as a key step in transcriptional regulation for many genes, during development and in stem cells (Guenther *et al.* 2007; Muse *et al.* 2007; Zeitlinger *et al.* 2007). At paused loci, after initiation, RNA Pol II first passes through the promoter but then stops at ∼30–60 bp from the transcription start site (Kwak and Lis 2013). Productive transcription then requires release from the paused state. PPP is brought about by the negative transcription elongation factor (NELF) and 5,6-dichloro-1-β-d-ribofuranosylbenzimidazole (DRB)-sensitivity inducing factor (DSIF) protein complexes, which were identified as factors responsible for DRB sensitivity of transcription elongation (Wada *et al.* 1998; Yamaguchi *et al.* 1999). These complexes bind RNA Pol II and halt its progress downstream of the promoter. This pause is alleviated by a positive transcription elongation factor complex (P-TEFb) (Figure 1A), which consists of cyclin T and cyclin dependent kinase-CDK9 (Marshall and Price 1995). Once recruited to the paused complex, CDK9 phosphorylates NELF and DSIF leading to ejection of NELF from the paused complex while DSIF assists Ser-5 phosphorylated RNA Pol II in productive elongation (Jonkers and Lis 2015). The PTEFb complex is, in turn, regulated through sequestration by 7SK snRNP complex. P-TEFb is required for release paused RNA polymerase II into productive elongation (Kwak and Lis 2013). Sequestration of P-TEFb by 7SK snRNP leads to its unavailability for mediating pause release, which, in turn, regulates transcription elongation via sustained pause of RNA Pol II. Mammalian 7sk-snRNP complex consists of 7sk RNA, Hexim1/2, Larp7, and MePCE. *Drosophila* homologs of components of mammalian 7sk-snRNP complex were identified and characterized recently (Nguyen *et al.* 2012). These include Bin3 (MePCE ortholog), Larp (Larp7 ortholog), Hexim (HEXIM1/2 ortholog), and d7SK RNA. All are highly conserved at functional levels with their mammalian counterparts. Signaling events of pathways such as ERK, TCR, etc., trigger liberation of P-TEFb. Thus, making sequestration and liberation of P-TEFb a context dependent process that is critical for regulating expression of gene regulation depending on the context.

**Figure 1:**
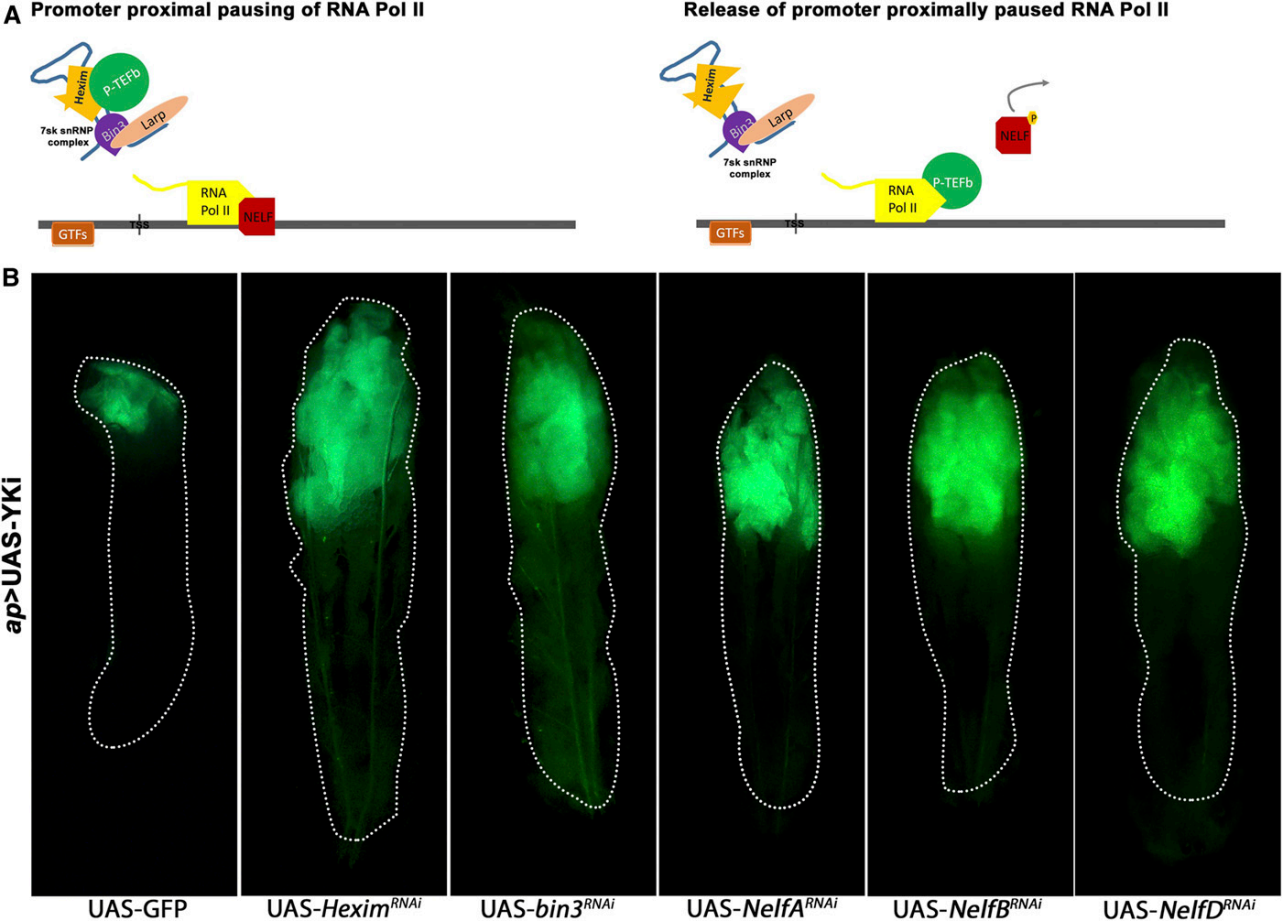
Identification of complexes involved in promoter proximal pausing as tumor suppressors. (A) A schematic representing known function of two complexes we identified as candidate tumor suppressors. The 7SK snRNP complex regulates promoter proximal pausing by sequestring the P-TEFb complex, while the NELF complex is involved in the formation of a stall of RNA Pol II at the promoter proximal region. As dictated by surrounding cues, P-TEFb is released by the 7SK snRNP complex. Thus, freed P-TEFb is recruited to stalled RNA Pol II, where it brings about release of RNA Pol II from the paused state. (B) Larval images showing wing imaginal discs expressing GFP at low magnification. Dimensions of GFP-expressing tissue is indication of growth in imaginal discs. Top row: Larvae overexpressing only Yki (crossed to UAS-GFP as control) and those in combination with RNAi-mediated knockdown of 7sksnRNP components: *Hexim* or *bin3* using GAL80^TS^; *ap*-GAL4; UAS-GFP. Bottom row: wing discs overexpressing Yki in combination with RNAi-mediated knockdown of NELF components (from left to right) *NelfA*, *NelfB*, and *NelfD* (also known as TH1) using GAL80^TS^; *ap*-GAL4; UAS-GFP. Note significantly larger GFP expressing-wing discs (green) in larvae that are overexpressing Yki and also depleted for a component of PPP.

Interestingly, CDK9 has been shown to be important for transcription of target genes of oncogenes such as Myc (Kanazawa *et al.* 2003) and YAP (Galli *et al.* 2015). Here, we present evidence of tumor suppressor function of various components involved in PPP, specifically in the context of elevated Yki activity. Our findings show that factors involved in PPP and its regulation are important to restrict Yki driven growth and to prevent neoplastic transformation *in vivo*.

## Material and Methods

### *Drosophila* strains

The following *Drosophila* strains are used in this study: *ap-Gal4* (Cohen *et al.* 1992) and *UAS-Yki* (Huang *et al.* 2005). The following RNAi stocks were obtained from the Vienna *Drosophila* RNAi Center and Bloomington *Drosophila* stock Center: *UAS-NelfA^RNAi^* (KK106245, TRiP #32897), *UAS-NelfB^RNAi^* (KK108441, TRiP #42547), *UAS-NelfE^RNAi^* (TRiP # 32835), *UAS-NelfD^RNAi^* (KK100009, TRiP # 38934, #42931), *UAS-bin3*^*RNAi*^ (KK101090, TRiP #41527), *UAS-Hexim*^*RNAi*^ (KK100500). *UAS-CDK9* was obtained from FlyORF (#F001571).

### Spatio-temporal regulation of transgene expression in wing imaginal disc

The *apterous* enhancer was used to drive expression of *Gal4* conditionally in dorsal compartment of wing imaginal discs. *Gal4* activity was regulated using Gal80^TS^, which allows expression of transgenes at permissive temperature of 29° as against restrictive 18° temperature. In all experiments, *tubulin*-Gal80^TS^ was used. *Drosophila* crosses were allowed to lay eggs for 3 days at 18°, and were then flipped or discarded. Larvae were then allowed to grow for additional 5 days before switching to temperature of 29°. At 29° they were maintained for 4–14 days. All crosses were using *tubulin*-GAL80^TS^; *ap*-GAL4; UAS-GFP. Thus, all experimental crosses had one copy of GFP, while control crosses had two copies of GFP. Detailed methodology is provided in Groth *et al.* (2020). Larval images were taken in bright field and in GFP channel with a Leica stereomicroscope. Image processing was done using Adobe Photoshop 6 and ImageJ.

### Immunohistochemistry

The following primary antibodies were used: rat anti-Ecadherin, mouse anti-MMP1 (Developmental Studies Hybridoma Bank). Rhodamine-phalloidin (ThermoFisher Scientific, Cat no R415) was used to stain actin in tissue.

Third instar larvae were dissected in PBS. Samples were fixed in 4% PFA for 20 min, followed by three 10-min washes in PBT (PBS-Tween20) at room temperature. Then, 5% BSA in PBS was used for blocking followed by overnight incubation in primary antibody at 4°. Next day, the samples were washed with PBT, three times for 10 min each followed by incubation with secondary antibody for 2 hr at room temperature. Samples were then washed with PBT and stained for DNA using 4′,6-diamidino-2-phenylindole (DAPI; Sigma Aldrich) for 5 min. Wing disc tissue was then mounted on slides in Antifade Gold mountant (ThermoFisher Scientific). Imaging was done on a Leica SP8 confocal laser-scanning microscope. Image processing was done using ImageJ and Adobe Photoshop 6. Measurement MMP1 intensities and comparison between different genotypes was carried out using ImageJ, statistical analysis (one-way ANOVA) was done using Prism-Graphpad 5.

### RNA-seq

Induction procedure for transgenes was followed as mentioned earlier. Wing imaginal disc tissue was dissected on 4th–5th day after induction for *ap > GFP*, *ap > UAS-Yki*, *ap > Nelf-A RNAi* (KK106245), *ap > UAS-Yki*, *UAS Nelf-A RNAi*. Larvae were washed in RNase-, DNase-free ultrapure water (GiBCO), and then dissections were done in RNase-, DNase-free PBS (GiBCo). Number of wing imaginal discs collected was 150, 70, 150, 25, respectively for *ap > GFP*, *ap > UAS-Yki*, *ap > Nelf-A RNAi* (KK106245), *ap > UAS-Yki*, and *UAS Nelf-A RNAi*. Collection was done in TRiZOL reagent (ThermoFisher Scientific). Each genotype was collected in three biological replicates. RNA sequencing was done on an Illumina platform.

### RNA-seq data analysis

RNA-seq analysis was performed using the HISAT 2.0 package protocol as explained in Pertea *et al.* (2016). To identify significantly differentially expressing genes in different combinations of comparisons, DEseq package and EdgeR were used (Anders and Huber 2010). The entire RNA-seq data set is available on GEO database (https://www.ncbi.nlm.nih.gov/geo/query/acc.cgi?acc=GSE151935).

The list of genes obtained was then used as input for the web-based tool venny (http://bioinfogp.cnb.csic.es/tools/venny/index.html) to obtain a list of genes that are unique to each genotype, overlapping between all three or combination of any two genotypes.

### Gene ontology analysis

For gene ontology (GO) and pathway enrichment analysis, we utilized STRING10 (Szklarczyk *et al.* 2017). We used gene lists that are significantly differentially expressed in single genotype or a combination of genotypes as mentioned in the results section, as input to the STRING. The output files were downloaded as interaction network and list of genes from input that are enriched in different GO categories or as KEGG pathways.

### Data availability

The authors state that all data necessary for confirming the conclusions presented in the article are represented fully within the article. All *Drosophila* stocks are available upon request. RNA-seq data are available at GEO with the accession number: GSE151935. Supplemental material available at figshare: https://doi.org/10.25386/genetics.12689318.

## Results

### Depletion 7SK snRNP complex components cooperates with Yki in causing tumorous growth

Studies using *Drosophila* tumor models have found that larvae containing proliferating tumors are unable to enter pupariation and continue to grow (Gateff *et al.* 1993). The resulting giant larva phenotype can be used in genetic screens to identify tumor-causing genotypes. We made use of this property to identify candidate genes in a genetic screen for tumor suppressors cooperating with Yki [the entire screen is published elsewhere (Groth *et al.* 2019)]. We found that RNAi-mediated depletion of *bin3* or *Hexim*, components of the 7SK snRNA complex in combination with Yki overexpression led to massive overgrowth in wing disc tissue and giant larval phenotype (Figure 1B). Wing discs expressing Yki alone show only moderate overgrowth phenotype, and larvae eventually pupate (Figure 1B). Depletion of 7SK snRNP components did not produce overgrowth on their own (Supplemental Material, Figure S1), but only did so when coupled with Yki overexpression. We also did not observe wing disc overgrowth when depletion of 7SK snRNP components in combination with overexpression of other well-known oncoproteins such as epidermal growth factor receptor (EGFR) or notch intracellular domain (NICD) (Figure S2). Thus, our observations suggest that, *Drosophila* 7SK snRNP complex may function, specifically, to repress tumorigenic potential of Yki *in vivo* in an epithelial tumor model.

### Components of the NELF complex may function as tumor suppressors

The NELF complex is composed of four subunits: NELF-A, -B, -C/-D and -E. Depletion of each of the NELF components using RNAi in combination with Yki also produced a giant larval phenotype (Figure 1B) and massively overgrown wing disc tissue compared to the larvae overexpressing only Yki (Figure 1B). Depletion of the NELF components on their own did not cause such giant larval phenotype or overgrowth of the wing disc tissue (Figure S1). These components too did not show any tumor phenotype in the context of overexpressed EGFR or NICD (Figure S2).

It was intriguing to find multiple components of the two spatio-temporally separated protein complexes, involved in the regulation of transcription elongation, among the tumor suppressors identified in a genome-wide screen for factors cooperating with Yki in growth regulation (Groth *et al.* 2019).

### Neoplastic transformation induced by Yki combined with depletion of 7SK snRNP or NELF complexes

Yki is known to promote cell proliferation and cell survival. Thus, it is possible that larger size of the wing disc tissue observed upon loss of either 7SK snRNP or NELF complex is a result of enhancement of growth and survival effect of Yki, and not a neoplastic transformation. To distinguish between the two possibilities, we analyzed the tumor tissue using markers that indicate neoplastic transformation.

**Figure 2:**
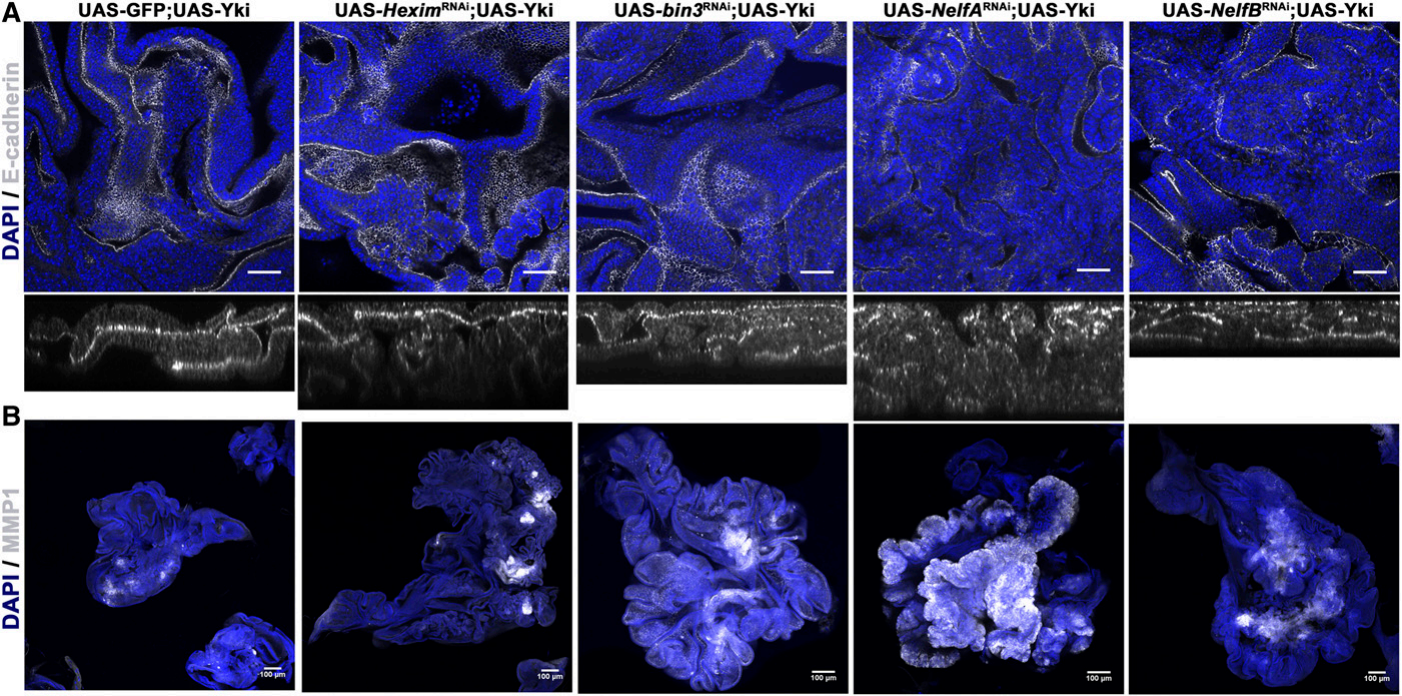
Characterization of tumors induced in the wing disc. (A) Disruption of characteristic epithelial apico-basal polarity in tumor discs. Images of wing discs overexpressing Yki alone (crossed to UAS-GFP as control) or in combination with RNAi-mediated knockdown of *Hexim*, *bin3*, *NelfA* or *NelfB* using GAL80^TS^; *ap*-GAL4; UAS-GFP (Bar, 10 µm). Discs are stained for E-Cadherin (white) expression and localization. Bottom panel of each image shows orthogonal optical section of respective genotype. Note delocalization of E-Cad in tumorous tissues caused by the depletion of a component of PPP and Yki overexpression (higher magnification images are shown for few genotypes). All discs are also stained with DAPI (blue) to visualize nuclei. (B) Increased expression of MMP1 is observed in tumor discs. Images of wing discs overexpressing Yki alone (crossed to UAS-GFP as control) or in combination with RNAi-mediated knockdown of *Hexim*, *bin3*, *NelfA*, or *NelfB* using GAL80^TS^; *ap*-GAL4; UAS-GFP (Bar, 100 µm). Wing discs are stained for MMP1 (white). Note increased MMP1 staining in tumorous tissues caused by the depletion of a component of PPP and Yki overexpression. All discs are also stained with DAPI (blue) to visualize nuclei.

First, we examined epithelial cell polarity. Neoplastic transformation of an epithelial tissue is accompanied by the loss of their characteristic apico-basal cell polarity. E-cadherin (E-Cad) is a subapically localized protein that provides a convenient marker for epithelial polarization (Tanos and Rodriguez-Boulan 2008). Wing discs overexpressing Yki alone showed localization of E-Cad, in a pattern similar to the wild-type wing discs, although the former discs are much larger (Figure 2A). This indicated that Yki overexpression caused overgrowth of the epithelium without perturbation of epithelial cell polarity. In contrast, when Yki overexpression was combined with depletion of a component of the 7SK snRNP complex or the NELF complex, subapical localization of E-cad was lost or perturbed (Figure 2A). Additionally, we analyzed F-Actin, which localizes near the apical junctions of the wing disc epithelial cells, using rhodamine-labeled phalloidin. As with E-Cad, we observed loss of apical localization of F-actin in the Yki expressing tissue depleted of a component of the 7SK snRNP or the NELF complex, but not in wing disc tissue expressing Yki alone (Figure S3). We did not observe any change in cell polarity, as indicated by E-Cad or F-Actin localization in wing discs with depletion of components of 7SK snRNP and NELF complexes alone (Figure S4A; data not shown for F-Actin).

The matrix metallo-protease MMP1 has been used as a marker of epithelial to mesenchymal transition (EMT) and neoplastic transformation in *Drosophila* tumor models. MMP1expression is elevated in tumor models and its depletion by RNAi has been reported to block metastasis (Uhlirova and Bohmann 2006; Beaucher *et al.* 2007; Miles *et al.* 2011). We examined the effects of depleting components of 7SK snRNP and NELF complexes in Yki-expressing tissue on the levels of MMP1 by immunohistochemistry. We observed significantly elevated levels of MMP1 in wing discs overexpressing Yki and depleted for Bin3, Hexim, or the NELF complex (Figure 2B, Figure S5A). We observed only marginal increase (statistically insignificant) in MMP1 levels in wing discs expressing Yki alone (Figure 2B, Figure S5A). We did not observe any detectable change in the intensity of MMP1 levels in the wing discs depleted for the components of 7SK snRNP and NELF complexes alone (Figure S4B and Figure S5B).

Taken together, tumors formed upon depletion of 7SK snRNP or NELF complex components in combination with Yki exhibit neoplastic characters. As neither genetic change alone produced these results, it appears that they act in combination to promote neoplasia, a classical mechanism of cooperative tumorigenesis as known in mammals. These observations provide evidence that the activity of 7SK snRNP and NELF complexes may have a tumor-suppressing function, but only in the context of elevated Yki activity.

### CDK9 is required for Yki-mediated tumorigenesis

**Figure 3:**
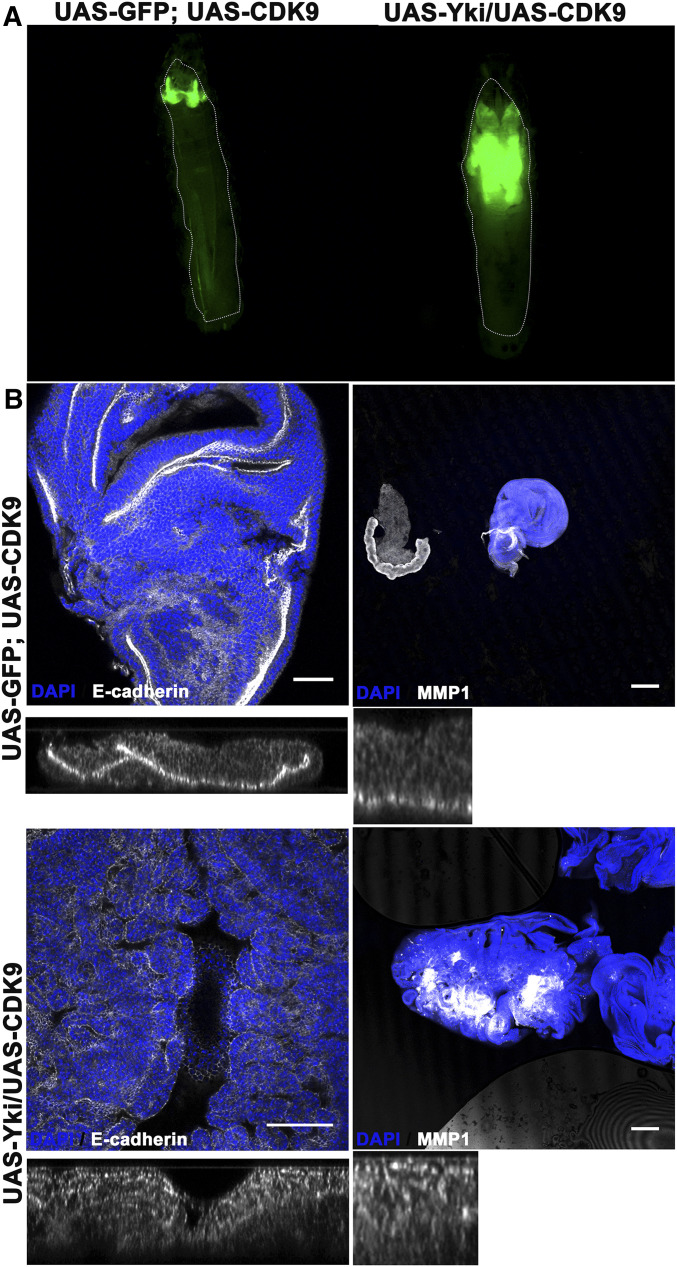
CDK9 cooperates with Yki in tumorigenesis. (A) Larval images showing growth observed in combination of UAS-CDK9 and UAS-Yki as compared to UAS-CDK9 alone (crossed to UAS-GFP as control) using GAL80^TS^; *ap*-GAL4; UAS-GFP. The combined overexpression phenocopies the phenotypes observed in Figure 2B. (B) Characterization of tumor tissue caused by combined overexpression of CDK9 and Yki using GAL80^TS^; *ap*-GAL4. Top row of images shows wing disc tissue overexpressing CDK9 alone, while the bottom row shows combined overexpression of CDK9 and Yki. Discs in the left column are stained for E-Cadherin (white) (Bar, 10 µm) and those in the right column are stained for MMP1 (white) expression (Bar, 100 µm). Please note deregulated E-cad localization (optical z-sections and two different magnification levels are shown below the discs) and increased MMP1 expression in tissues that overexpress both CDK9 and Yki, suggesting their neoplastic tumor state. All discs are also stained with DAPI (blue) to visualize nuclei. Both the discs stained for MMP1 are imaged at lower magnification (10X) for better comparison, as tumorous disc is too large to show at higher magnification.

The 7SK snRNP and NELF complexes help in maintaining the paused state of RNA Pol II. Our findings raised the question of whether pausing of RNA Pol II *per* *se* served to limit the tumor promoting potential of Yki activity. If this is the case, we reasoned that using an alternative means to release RNA Pol II should also lead to tumorigenesis in the context of Yki overexpression. The P-TEFb complex, comprising cycT/CDK9, is required for release of paused RNA Pol II and effective elongation of mRNA. CDK9 phosphorylates the NELF complex, leading to eviction of NELF from the pause site. This in turn facilitates release of paused RNA Pol II, aiding in productive elongation. CDK9 also acts directly on RNA Pol II, phosphorylating it on S5 in the C-terminal domain, a known mark of elongating RNA Pol II (Jennings 2013). As the P-TEFb complex is normally rendered inactive through sequestration by 7SK snRNP complex, we hypothesized that overexpressing CDK9 might bypass normal regulation of pausing, leading to inactivation of NELF complex and RNA Pol II release. Consistent with this hypothesis, we indeed observed massive tissue overgrowth when Yki was co-expressed with CDK9, while overexpression of CDK9 alone did not cause any such phenotype (Figure 3A). Such overgrowth phenotype was not observed when CDK9 was overexpressed in the background of elevated activities of EGFR or Notch (Figure S2). This suggests that PPP-mediated regulation of growth is Yki-specific.

Wing discs expressing UAS-CDK9 together with UAS-Yki also showed loss of apically localized E-Cad as well as elevated MMP1 expression (Figure 3B), compared to tissue expressing UAS-CDK9 alone or UAS-Yki alone. This indicates neoplastic transformation in wing discs co-expressing Yki and CDK9, similar to the transformation caused by depletion of 7SK snRNP and NELF complex components in combination with overexpressed Yki.

**Figure 4:**
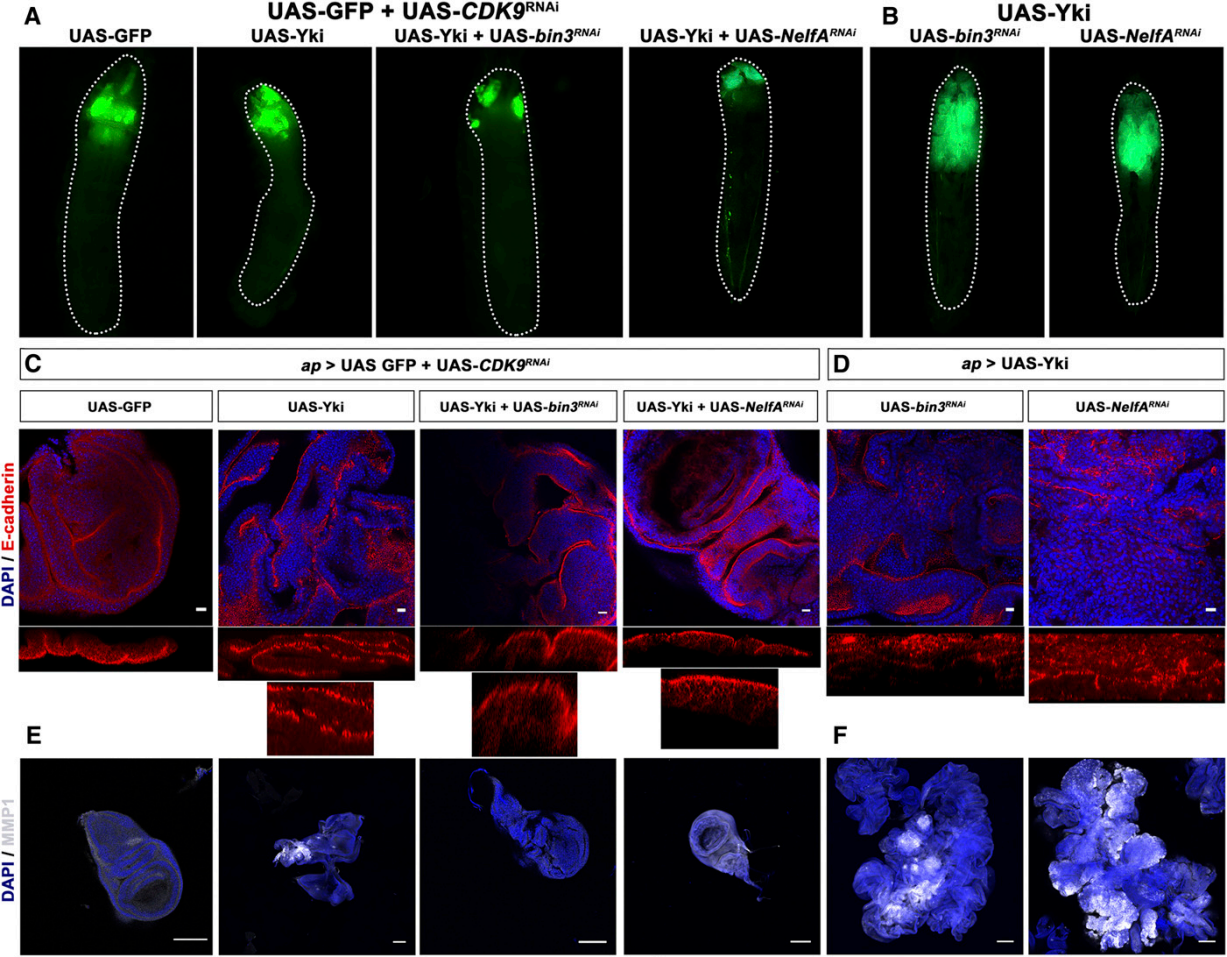
CDK9 is necessary for Yki-mediated tumorigenesis. (A) Loss of CDK9 rescues tumor phenotype. The images show GFP-expressing wing discs of various genotypes as indicated. Size of the wing discs may be discerned by the amount of larval space occupied by GFP-expressing tissue. RNAi-mediated depletion of *cdk9* inhibited tumor formation caused by a combination of overexpression of Yki and depletion of a component of the PPP. The GFP-marked wing tissue is of the same size as in controls. All crosses were using GAL80^TS^; *ap*-GAL4; UAS-GFP. (B) Tumorous wing disc phenotypes caused by the overexpression of Yki in the background of depletion of *bin3* or *NelfA* shown here again as a control to (A). (C) Restoration of apico-basal polarity in wing disc tissue. The images show wing discs of various genotypes as indicated stained for E-Cad (red). RNAi-mediated depletion of *cdk9* restored normal apical localization of E-Cad (optical z-sections are shown below the discs) in wing discs that overexpress Yki and are also depleted for a component of the PPP. All discs are also stained with DAPI (blue) to visualize nuclei (Bar, 10 µm). (D) Tumorous wing discs (stained for E-Cad) of larvae overexpressing Yki in the background of depletion of *bin3* or *NelfA* shown here again as a control to (C). (E) Restoration of MMP1 levels. The images show wing discs of various genotypes as indicated stained MMP1 (white). RNAi-mediated depletion of *cdk9* restored normal levels of MMP1 in wing discs that overexpress Yki and also depleted for a component of the PPP. All discs are also stained with DAPI (blue) to visualize nuclei (Bar, 100 µm). (F) Tumorous wing discs (stained for MMP1) of larvae overexpressing Yki in the background of depletion of *bin3* or *NelfA* shown here again as a control to (E).

As further test of this model, we asked whether CDK9 is essential for tumorigenic cooperation between depletion of 7SK snRNP complex components and Yki. Depletion of *cdk9* effectively suppressed the tissue overgrowth caused by depleting *bin3* or *Hexim* in Yki expressing tissue (Figure 4A). Those wingdiscs also showed normal apical localization of E-Cad and wildtype levels of MMP1 expression, suggesting complete suppression of tumorous growth (Figure 4, B and C).

Given that CDK9 is known to act directly on both NELF proteins and RNA Pol II, we wondered whether CDK9 activity would be required in the absence of the NELF complex. As shown above in the case of removing the 7SK snRNP complex, depletion of *cdk9* suppressed overgrowth caused by RNAi-mediated depletion of *NelfA* and overexpression of Yki (Figure 4A). This was accompanied by restoration of apico-basal polarity and MMP1 expression to wild-type levels (Figure 4, B and C). This finding provides evidence that alleviation of pausing by removal of NELF complex is not sufficient without CDK9 activity. This presumably reflects an importance of activation of RNA Pol II by CDK9-mediated phosphorylation.

**Figure 5:**
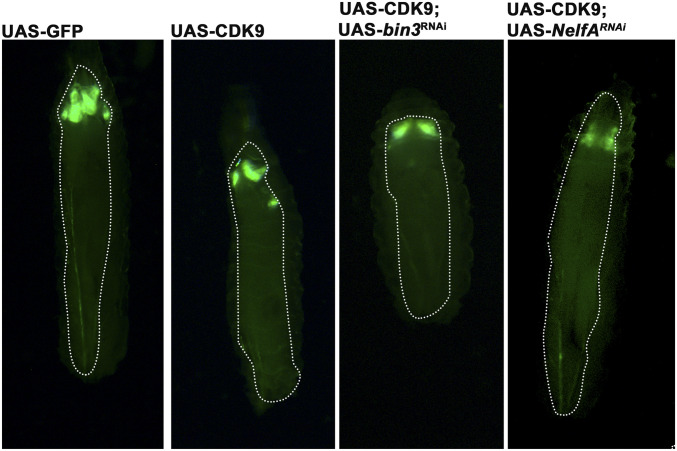
Yki is the driver of tumorigenesis. Larval images showing phenotype of UAS GFP in combination with (left to right) UAS-GFP, UAS-CDK9 followed by UAS-CDK9 and UAS-*bin3*^RNAi^, UAS-CDK9, and UAS-*NelfA*^RNAi^. None of them show overgrowth phenotype as observed when Yki is overexpressed, suggesting CDK9 may induce tumorous growth only in the context of overexpressed Yki. All crosses were using GAL80^TS^; *ap*-GAL4; UAS-GFP.

We then examined if depletion of the complexes associated with PPP and increased CDK9 levels are sufficient to cause overgrowth phenotype, or whether the growth is tightly coupled to the presence of a growth driver such as Yki. Depletion of components of 7SK snRNP or NELF complexes in the background of overexpressed CDK9 did not cause any growth phenotype or morphological alteration in wing disc epithelium (Figure 5). This suggests that deregulation of RNA Pol II pausing is not sufficient on its own to produce an overgrowth or neoplastic phenotype; yet it does so in the context of Yki overexpression. In the context of elevated Yki activity, there appear to be two brakes, each of which must be removed by CDK9 activity to allow excess Yki to produce tumors in *Drosophila* wing disc tissue.

### Tumorigenesis induced by alleviation of pausing is associated with deregulated proteostasis

As overexpression of Yki was essential, although not sufficient, to cause neoplastic tumors, genetic experiments above provided an opportunity to distinguish between Yki-activated genes that cause simple hyperplastic growth of the discs (when Yki is overexpressed in a wild-type background) *vs.* causing neoplastic growth (when Yki is overexpressed along with depletion of *bin3*, *Hexim*, or NELFs).

**Figure 6:**
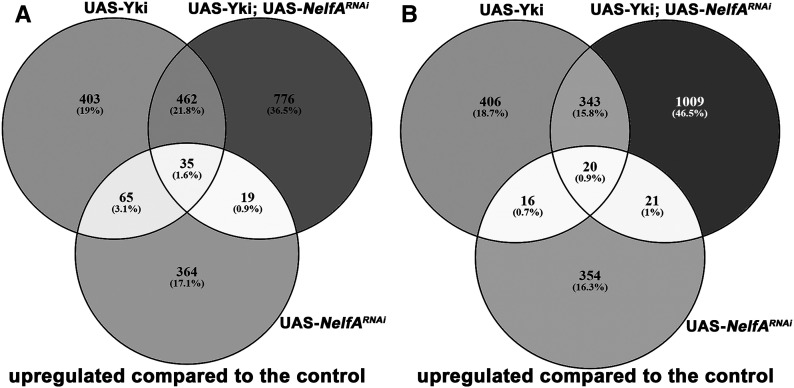
Identification of genes potentially involved in Yki-mediated tumorigenesis. (A) Venn diagram showing number of common and unique genes, who expression is upregulated in comparison with *ap > GFP* from different genotypes as indicated in the figure. (B) Venn diagram depicting number of common and unique genes downregulated in comparison with *ap** > GFP* from different genotypes.

We carried out RNA-seq to identify differentially expressed genes in discs depleted for *NelfA* and overexpressing Yki as well as both individual treatments. We also carried out RNA-seq for GFP expressing wild-type wing discs as a control. We find that transcripts corresponding to 776 genes were uniquely upregulated (Figure 6A) and 1009 genes were uniquely downregulated (Figure 6B) in the tumorous wing discs (*ap* > UAS-Yki; UAS-*NelfA^RNAi^*), compared to all other genotypes including wild-type discs (noncoding transcripts are not included in this estimation). When compared to the list of direct targets of Yki (reported by based on ChIP-seq data), we find 38 (4.9%) of the upregulated genes and 84 (8.3%) of the downregulated genes are presumptive direct targets of Yki (Table S1).

We also observed an enhancement of effect of Yki (compared to wildtype discs) in a subset of transcripts that were common to nontumorous tissue overexpressing Yki alone (*ap* > UAS-Yki) and tumorous *ap* > UAS-Yki; UAS-*NelfA^RNAi^* tissue. We reasoned that since PPP functions to attenuate expression of genes, identifying transcripts whose expression is further up- or downregulated in *ap* > UAS-Yki; UAS-*NelfA^RNAi^* tissue (compared to *ap* > UAS-Yki) may give a better indication of the role of PPP in Yki-mediated growth. We find that transcripts corresponding to 155 genes that are upregulated in both nontumorous *ap* > UAS-Yki discs and tumorous *ap* > UAS-Yki; UAS-*NelfA^RNAi^* discs, but degree of enhancement was higher in tumorous tissue. Likewise, these transcripts corresponding to 160 genes, whose expression was downregulated compared to wildtype discs, were common to both nontumorous and tumorous tissue, but degree of downregulation was higher in tumorous tissue. Interestingly, 31 (20%) of these upregulated genes (*n* = 155) and 35 (21.9%) of downregulated genes were presumptive direct targets of Yki, suggesting that we indeed have captured many targets of Yki that are regulated by PPP and misregulated due to RNAi medicated knockdown of many components of the pausing machinery.

We used genes corresponding to these transcripts to perform GO analysis in order to explore gene sets that show enrichment and might indicate pathways or processes that are involved in tumorigenesis. For this purpose, the STRING tool was utilized (Szklarczyk *et al.* 2017). STRING output is based on statistical enrichment score of interactions obtained from the input compared to a random set of genes from the genome of the organism, in this case *D. melanogaster*. STRING also collates data from manually curated databases of interactions such as Kyoto Encyclopedia of Genes and Genomes (KEGG) and GO terms.

We observed enrichment for pathways involved in ribosome and its biogenesis in the upregulated set (Table 1 and Figure 6). Interestingly, protein processing in endoplasmic reticulum, regulators of proteasome function, and different components of proteasome were enriched among genes downregulated in tumorous tissues (Table 2). These observations indicate overall deregulation of protein homeostasis (proteostasis) in tumors caused by depletion of *NelfA* in combination with Yki overexpression, consistent with recent data on human cancers (Ruggero 2013; Pelletier *et al.* 2017).

**Table 1:** List of genes whose expression is upregulated in the wing discs of *ap*-GAL4/UAS-*NelfA*^RNAi^; UAS-Yki

Aminoacyl-tRNA biosynthesis		Ribosome		Ribosome biogenesis in eukaryotes	
Gene name	logFC	Gene name	logFC	Gene name	logFC
Slimp	2.124626	RpL24-like	1.256082	Non1	2.292457
Aats-leu	1.413973	RpL5	1.202779	Ns2	0.900983
Aats-thr	0.912237	RpL15	1.130382	RIOK1	1.354352
Aats-cys	0.813931	mRpL28	0.969423	CG12301	0.997310
Aats-tyr-m	1.047768	mRpL9	0.799329	Bka	0.876333
Aats-pro	1.07342	RpS17	0.82042	eIF6	0.745744
Aats-ile	0.73741	mRpL35	0.997681	l(3)72Dn	0.800876
CG4573	1.138148	RpS23	0.782542	CG8064	0.778604
CG1750	1.487797	RpS4	0.775449	Nmd3	0.716537
CG6796	0.925494	RpL27A	0.716573	Mat89Ba	0.713426
CG7441	0.884721	RpL32	0.681588	CG11920	0.750235
CG17259	0.726080	RpL40	0.674508	CG3071	0.713535
Aats-trp	0.732224	RpS29	0.743389	CG33158	0.595732
Aats-asp	0.747097	RpL26	0.620538	CG13185	0.823244
Aats-gly	0.613889	mRpL10	0.692489	CG7246	0.798345
CG5463	1.037030	mRpL3	0.671734	CG8549	0.593618
Aats-ala-m	0.602770	RpL35	0.631348		
CG5660	0.663614	RpL27	0.594275		
		RpL28	0.629209		
		RpL21	0.600412		
		RpL22-like	1.081389		
		RpS3A	0.587288		
		RpL37A	0.3662		
		mRpL11	0.624297		

**Table 2:** List of genes whose expression is downregulated in the wing discs of *ap*-GAL4/UAS-*NelfA*^RNAI^; UAS-Yki

Proteasome		Protein processing in endoplasmic reticulum	
Gene name	logFC	Gene name	logFC
Rpn7	−1.084698	prtp	−1.46289
Rpn13	−0.949757	Sec61gamma	−1.65593
Rpn2	−0.900781	Sec61beta	−1.31964
Prosalpha3	−0.910536	CG5885	−1.28449
Rpn3	−0.850895	Sec61alpha	−1.25184
Rpn1	−0.879125	TRAM	−1.3381
Pomp	−0.795409	Pdi	−1.145
Prosalpha5	−0.834712	SsRbeta	−1.23571
Prosbeta4	−0.76685	Sec13	−1.01315
Prosbeta7	−0.717883	Sec63	−0.97489
Prosbeta2	−0.706638	CG14476	−1.03296
Prosbeta5	−0.687177	Sec24CD	−0.87522
Prosalpha4	−0.694159	Ostgamma	−0.97374
Prosbeta6	−0.631819	Ost48	−0.88256
Rpn10	−0.592037	CG4164	−1.21065
Rpn12	−0.597364	ergic53	−0.86625
		Plap	−0.86843
		OstStt3	−0.90274
		Gp93	−0.89197
		l(1)G0320	−0.88951
		CG33303	−0.8065
		Hsc70-3	−0.9493
		CG5510	−0.81474
		p47	−0.78553
		Crc	−0.86323
		CG6453	−0.81869
		Sec23	−0.73903
		ERp60	−0.76882
		Der-1	−0.80252
		Csp	−0.64369
		CaBP1	−0.61193
		CG1597	−0.67306

## Discussion

PPP has emerged as a critical regulatory step in gene expression (Core and Adelman 2019). It involves stalling of RNA Pol II 20–60 nucleotides downstream of the transcription start site, and controlled release of RNA Pol II when triggered by signals from the surroundings. Many studies in recent years have elucidated mechanisms by which RNA Pol II is stalled and the factors that bring about pausing as well as release of the paused RNA Pol II. Our *in vivo* model for tumorigenesis has allowed us to elucidate the functions of the NELF, 7SKsnRNP, and P-TEFb complexes in the context of growth control *in vivo*. Previous studies have implicated NELF in regulating the response of embryonic stem cells to signaling cues such as fibroblast growth factor (FGF; Williams *et al.* 2015). Furthermore, PPP has been shown to be important for coordination of expression genes involved in morphogenesis of *Drosophila* embryo (Lagha *et al.* 2013). Our findings provide direct evidence that PPP can limit tumor formation in the context of the Hippo tumor suppressor pathway. Depletion of these factors alone, or even in combination with overexpression of CDK9, was not sufficient to induce tumorous growth but did so when combined with overexpression of Yki. This cooperation appears to be specific to Yki-induced tumors as there was no cooperation with other oncogenic drivers such as EGFR or activated Notch in wing disc tumor models. This suggests that pausing plays a previously unappreciated role regulating the output of Hippo pathway in growth control, thereby limiting its tumorigenic potential.

We were intrigued by the finding that CDK9 activity is required for Yki-driven tumor formation, even when the upstream and downstream pausing complex factors have been removed. These observations suggest that CDK9 activity is required not only to remove the “brake” exerted by NELF pausing complex, but also required to increase RNA Pol II activity through direct phosphorylation. Neither alone is sufficient. This suggests an overlapping “belt and suspenders” regulation to ensure that expression of Yki targets is maintained at appropriate levels for normal growth control, while preventing overexpression, which may lead to tumorigenesis. A mechanism of this sort allows for the possibility that other growth regulatory or metabolic homeostasis pathways might impact on the outcome of Yki activity via regulation of the CDK9. Indeed, evidence of a role for CDK9 in YAP/TAZ-mediated cell growth via regulation of a subset of YAP/TAZ target genes in mammalian liver cells has been demonstrated (Galli *et al.* 2015). Inhibition of CDK9 activity using flavopiridol nullified the effect of YAP S127A mutant form (the constitutively active form of YAP) on the expression of YAP target genes studied (Galli *et al.* 2015). Although this observation is not validated in fly tissues, perhaps PPP (including 7skRNP-, CDK9-, and NELFs)-dependent regulation of Yki is independent of the phosphorylation status of Yki, which implies a parallel function for PPP rather than it being upstream of Yki.

Our genetic model is also useful to study the importance of PPP in attenuating transcriptional output at genome wide scale. Preliminary observations of data generated by RNA-seq suggest that most genes that are differentially expressed when Yki is overexpressed show further changes in the same direction (up or down regulation) in combination of Yki overexpression with depletion of Nelf-A. Furthermore, we also report deregulation of proteostasis uniquely in tumor tissue. This is consistent with recent reports that deregulation of translation and deregulation of protein processing are important factors in progression of cancers and might be target for therapy (Ruggero 2013; Pelletier *et al.* 2017).

To conclude, our study has highlighted additional regulatory module on Yki driven tumorigenic activity, which impinges directly on transcription. It will be interesting to see the role of the PPP machinery, which has been reported to be highly conserved from *Drosophila* to humans (Peterlin and Price 2006), in the context of highly conserved Hippo pathway effectors YAP/TAZ. Considering the reported function of CDK9 in YAP-driven transcription, and the therapeutic accessibility of CDK9 activity (Galli *et al.* 2015; Blake *et al.* 2019), it is critical to understand the function of 7SK snRNP and NELF complexes in this context.
